# Bedside ultrasound is a practical measurement tool for assessing muscle mass

**DOI:** 10.5935/0103-507X.20170071

**Published:** 2017

**Authors:** Diogo Oliveira Toledo, Débora Carneiro de Lima e Silva, Dyaiane Marques dos Santos, Branca Jardini de Freitas, Rogério Dib, Ricardo Luiz Cordioli, Evandro José de Almeida Figueiredo, Silvia Maria Fraga Piovacari, João Manoel Silva Jr.

**Affiliations:** 1 Intensive Care Unit, Hospital Israelita Albert Einstein - São Paulo (SP), Brazil.

**Keywords:** Ultrasonography, Quadriceps muscle/diagnostic imaging, Body composition, Evaluation, Point-of-care testing, Intensive care units, Ultrassonografia, Músculo quadríceps/diagnóstico por imagem, Composição corporal, Avaliação, Testes imediatos, Unidades de terapia intensiva

## Abstract

**Objective:**

To evaluate the intra- and inter-reliability and the ease of measuring the quadriceps muscle thickness using bedside ultrasound.

**Methods:**

This is a prospective, observational study. The assessment of quadriceps muscle thickness was performed at two reference points and was quantified using portable B-mode ultrasound in two healthy volunteers. For standardization of measurements and validation of image collections, the team was trained through theoretical and practical classes, with a 6-hour workload.

**Results:**

A total of 112 images were examined by the coach and compared with the trainees. Pearson's correlation analysis found an excellent relationship between the coach and all trainees (R^2^ > 0.90). The best association was between the coach and the dietitians (R^2^: 0.99; p < 0.001), and the worst association was between the coach and the medical trainees (R^2^: 0.92; p < 0.001). In the Bland-Altman comparison, the highest error rate found between coach and trainees was 5.12% (95% confidence interval [CI] 3.64-12.37), and the lowest was 1.01% (95%CI 0.72 - 2.58); the highest bias of the values described was -0.12 ± 0.19, and the lowest was -0.01 ± 0.04.

**Conclusion:**

The data analyzed showed a good correlation between the measurements made by the coach and trainees, indicating that ultrasound of the quadriceps muscle is a viable and easily applicable tool.

## Abstract (pt)

**Objetivo:**

Avaliar a intra e interconfiabilidade, e a facilidade de medir a espessura muscular do quadríceps, usando ultrassom à beira do leito.

**Métodos:**

Trata-se de um estudo prospectivo, observacional. A avaliação da espessura muscular do quadríceps foi realizada em dois pontos de referência e quantificada com ultrassom portátil em modo B em dois voluntários saudáveis. Para padronização das medidas e validação das coletas das imagens, foi realizada capacitação da equipe por meio de treinamentos com aulas teóricas e práticas, com carga horária de 6 horas.

**Resultados:**

Foram examinadas 112 imagens pelo treinador e comparadas com os alunos. A correlação de Person encontrou excelente relação entre o treinador e todos os alunos (R^2^ > 0,90). A melhor associação foi entre o treinador e os nutricionistas (R^2^: 0,99; p < 0,001), e a pior, entre o treinador e alunos médicos (R^2^: 0,92; p < 0,001). Quanto à comparação de Bland-Altman, a maior porcentagem de erro encontrada entre treinador e alunos foi de 5,12% (IC95% 3,64 - 12,37) e a menor, 1,01% (IC95% 0,72 - 2,58); o maior viés dos valores descrito foi -0,12 ± 0,19, e o menor, -0,01 ± 0,04.

**Conclusão:**

Os dados analisados mostraram boa correlação entre as medidas feitas pelo instrutor e alunos, mostrando que o ultrassom de músculo quadríceps é uma ferramenta viável e de fácil aplicabilidade.

## INTRODUCTION

In critically ill patients, immobilization, sepsis, organ failure, and systemic inflammatory response are strongly related to muscle loss. Myopathy in critical patients is estimated to affect between 25 and 100% of intensive care unit (ICU) patients, depending on the permanence and the instrument used in the evaluation; in addition, it is an independent predictor of patient morbidity and mortality and of the loss of functional autonomy in the long term.^(1-3)^

The syndrome clinically described as weakness acquired in the ICU is associated with prolonged ventilatory weaning, rehabilitation impairment, longer hospital stay, and mortality. The risk stratification of patients with muscle loss is vital for optimizing clinical management, including motor rehabilitation and nutritional strategy, among others.^(4)^ Given the impact of this syndrome on clinical outcomes, recent research has focused on non-invasive methods that measure muscle thickness.^(5)^

Recently, new research has found that ultrasound measurements of the quadriceps muscle appear to be as accurate as those of computerized tomography and dual-energy X-ray absorptiometry (DEXA), which are the gold standards for the evaluation of muscle mass.^(2,6)^

Gruther et al. have shown that ultrasound is a valid and practical measurement tool for documenting muscle mass as part of the daily routine in the ICU. Moreover, they showed that those patients who presented greater losses of muscle mass remained longer in the ICU and that this loss appeared to be greater in the first weeks of immobilization.^(7)^ The study by Parry et al. evaluated the relationship of loss of lean mass by ultrasound with reduced strength and functionality, which may remain for years after dehospitalization.^(8)^

Ultrasound was also able to identify both morphological and structural alterations early in septic patients. In the same study, ultrasound was able to identify alterations that could be detected by more invasive methods, such as biopsy and electromyography.^(9)^

The usefulness of ultrasound has become the center of attention to monitor muscle evolution in severe patients because it is a non-invasive technique, highly practical and easy to apply at the bedside.^(1,2)^

Before validating the assessments of muscle mass loss made by ultrasound, we must demonstrate the reliability of the ultrasound measurements made by the coach compared to those made by himself and those made by the coach compared to those made by the trainees. Thus, the objective of this study was to train a multidisciplinary team to evaluate and validate the reliability of measurements made by trainees with no previous experience compared with measurements made by the coach.

## METHODS

This is a prospective, observational study performed in a tertiary hospital, approved by the Ethics Committee of the *Hospital Israelita Albert Einstein*, to evaluate the measurement of quadriceps muscle thickness (QMT), previously validated by Campbell et al.,^(10)^ performed on two healthy volunteers.

The QMT was quantified with a portable B-mode ultrasound in two healthy volunteers, one female and one male, who were lying in the supine position, with extended and relaxed knees. The male volunteer had a body mass index (BMI) of 23.5kg/m^2^ and an age of 35 years; the female volunteer had a BMI of 22kg/m^2^ and an age of 45 years.

**Figure 1:**
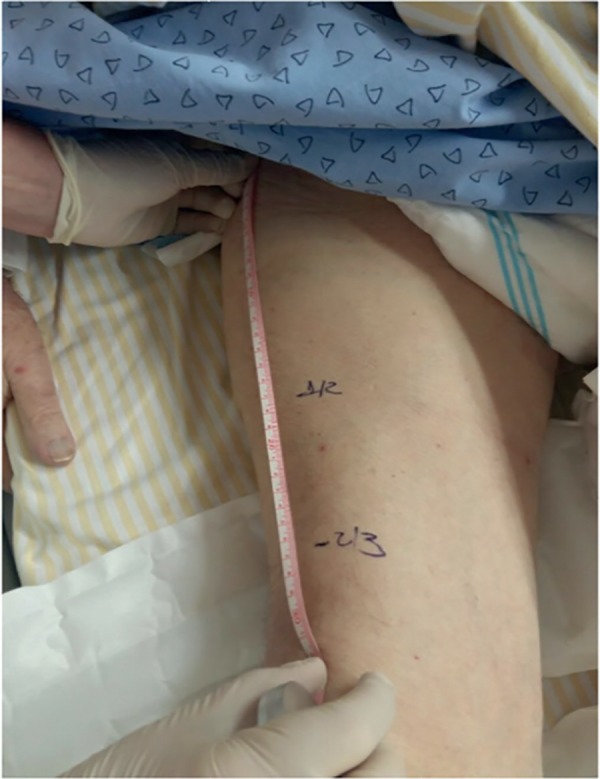
Reference points for measuring the thickness of the quadriceps muscle.

The QMT assessment was performed at two reference points identified in each quadriceps. The first point was marked on the anterior one-third of the superior iliac crest (ASIC) and the upper part of the patella, and the second point was identified at the midpoint between the ASIC and the upper part of the patella (Figure 1).

**Figure 2:**
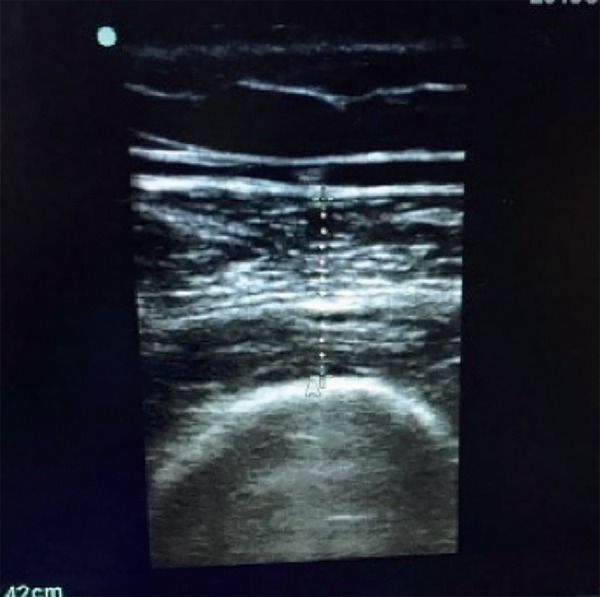
Quantification of muscle thickness.

Muscle thickness was quantified with a marking on the screen between the distance from the upper margin of the femoral bone and the lower border of the deep fascia of the rectus femoris muscle (Figure 2). Measurements with and without muscle compression were performed, and the QMT value in the right and left legs was the mean of the four readings performed on the right and left legs (two in each location).

For standardization of measurements and validation of image collections, the team was trained through theoretical and practical classes, with a 6-hour workload. The coach was a physician with advanced training on ultrasound at the bedside, and the group of trainees was composed of three dietitians, three physicians, and a physiotherapist, most without previous ultrasound experience. To validate the ultrasound images, the measurements were performed comparing the images made by the trainees with those made by the coach in the two healthy volunteers.

All of the measures performed by the trainees were correlated with the coach's measurements using Pearson's correlation coefficient, and the agreement analysis employed the Bland-Altman method. All data were entered into a spreadsheet (Microsoft Excel, Microsoft, Redmond, WA, USA) and were subsequently analyzed with the statistical software Statistical Package for Social Science (SPSS), version 20.0 (IBM Corp., Armonk, NY, USA), and MedCalc version 13.2.0 (MedCalc Software, Ostend, Belgium).

## RESULTS

**Figure 3:**
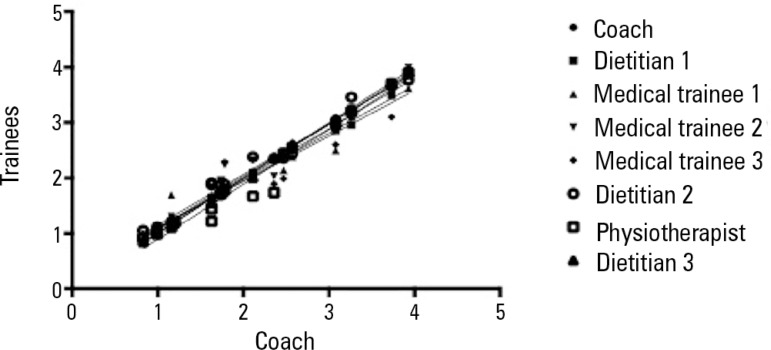
Correlation between coach and trainees.

**Figure 4:**
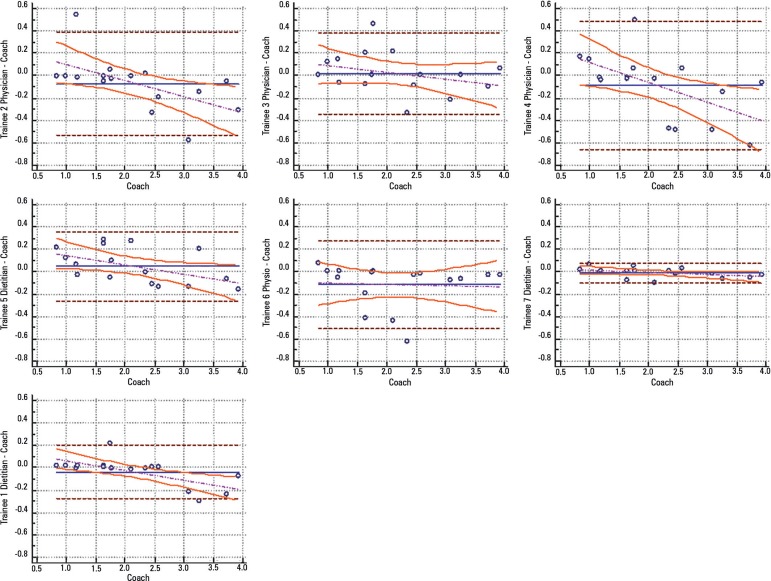
Agreement between coach and trainees - Bland-Altman.

The results of 112 images were examined by the coach and compared with those made by the trainees. Pearson's correlation analysis found an excellent relationship between the coach and all trainees (R^2^ > 0.90) (Figure 3). The best association was between the coach and the dietitians (R^2^: 0.99, p < 0.001), and the worst association was between the coach and the medical trainees (R^2^: 0.92; p < 0.001) (Table 1). Regarding the Bland-Altman comparison, the highest error rate found between the coach and the trainees was 5.12% (95% confidence interval [CI] 3.64 - 12.37), and the lowest was 1.01% (95%CI 0.72 - 2.58); the highest bias of the values described was -0.12 ± 0.19, and the lowest was -0.01 ± 0.04 (Figure 4).

**Table 1:** Correlations between trainees from different health areas and the coach

	Coach *versus* physician 1	Coach *versus* physician 2	Coach *versus* physician 3	Coach *versus* dietitian 1	Coach *versus* dietitian 2	Coach *versus* dietitian 3	Coach *versus* physiotherapist
Pearson r							
r	0.975	0.982	0.958	0.995	0.988	0.999	0.979
95% confidence interval	0.93 - 0.99	0.95 - 0.99	0.88 - 0.99	0.98 - 0.99	0.97 - 0.99	0.99 - 1.0	0.94 - 0.99
R^2^	0.950	0.964	0.918	0.989	0.977	0.998	0.959
p value							
P (two-tailed)	< 0.0001	< 0.0001	< 0.0001	< 0.0001	< 0.0001	< 0.0001	< 0.0001

## DISCUSSION

This study contributes to the literature by demonstrating excellent correlations between ultrasound measurements performed by a coach and trainees to determine QMT in healthy volunteers. These results reflect the capacity to standardize training for various professionals, including dietitians, physicians and physiotherapists with no previous ultrasound experience, and make this procedure feasible to determine QMT.

Although ultrasound in the ICU is a more practical examination in the evaluation of muscle loss, when compared with computed tomography,^(7)^ some points are still uncertain. Current concerns with this method are focused on those patients with edema, whose tissue thicknesses and measurements may be altered.^(11)^ Future research should address these questions since edema may not influence measurements when applied to the probe's maximum compression technique to assess QMT.

Since the reliability of ultrasound measurements is achievable by trainees with no previous ultrasound experience and QMT is a reflection of overall muscle mass, the next step is to apply this methodology to determine the loss of total muscle mass in critically ill patients, as was evaluated in patients hospitalized with pulmonary disease.^(12)^

Once the surveys establish QMT measures as reliable and valid, ultrasound can be used to screen patients at risk for muscle loss acquired in the ICU, at admission, and during hospitalization. Furthermore, ultrasound can measure the effectiveness of the nutritional strategy, along with motor rehabilitation interventions aimed at delaying or reversing muscle loss, reducing patient morbidity and ICU stay.

Some limitations of this study should be considered: (a) the study was not performed in critically ill patients, but the objective was to prioritize the technique to be applied in ICU patients in the next step; (b) since we only used two healthy volunteers for evaluation, the correlation may not be the same when applied to critically ill patients; and (c) only the muscle thickness technique (QMT) was performed as described, and it was not compared with the cross-sectional area technique, which has also been validated for quadriceps evaluation.

## CONCLUSION

There was an excellent correlation between the measurements performed by the specialist and the trainees, indicating that ultrasound of the quadriceps muscle is a viable and easily applicable tool for all health professionals as a method of evaluating and monitoring muscle mass. Ultrasound has demonstrated great potential for the linear evaluation of patients with muscle loss in the intensive care unit.
